# Construction and Immune Strategy Optimization of a Vaccine Strain for Influenza A (H5N8) Subtype

**DOI:** 10.3390/v17040544

**Published:** 2025-04-08

**Authors:** Shuxia Zhang, Jing Tang, Liqi Liu, Hejiang Wei, Li Xin, Kang Xiao, Jinbo Xiao, Jie Dong, Zi Li, Hongyan Bai, Shuaixing Wang, Wenfei Zhu, Lei Yang, Shumei Zou, Dayan Wang

**Affiliations:** National Key Laboratory of Intelligent Tracking and Forecasting for Infectious Disease, National Institute for Viral Disease Control and Prevention, Chinese Center for Disease Control and Prevention, Beijing 102206, China; zhangsx@ivdc.chinacdc.cn (S.Z.); tangjing@ivdc.chinacdc.cn (J.T.); liulq@ivdc.chinacdc.cn (L.L.); weihj@ivdc.chinacdc.cn (H.W.); xinli@ivdc.chinacdc.cn (L.X.); xiaokang@ivdc.chinacdc.cn (K.X.); xiaojb@ivdc.chinacdc.cn (J.X.); dongjie@ivdc.chinacdc.cn (J.D.); lizi@ivdc.chinacdc.cn (Z.L.); baihy@ivdc.chinacdc.cn (H.B.); zhuwf@ivdc.chinacdc.cn (W.Z.); yanglei@ivdc.chinacdc.cn (L.Y.); zousm@ivdc.chinacdc.cn (S.Z.)

**Keywords:** H5N8 subtype, avian influenza virus, adjuvant, immunogenicity, vaccine

## Abstract

Multiple subtypes of avian influenza virus (AIV), including H5N1, H5N6, and H5N8 viruses, are currently co-circulating in wild birds and poultry and causing sporadic human infections. Vaccine development is essential for pandemic preparedness. In this study, we constructed a candidate vaccine virus (CVV) using reverse genetics (RG) based on the sequence of the first human-infected H5N8 subtype AIV, A/Astrakhan/3212/2020 (H5N8). We evaluated the immunogenicity of the rH5N8/PR8 vaccine strain in combination with Alum, ISA51, and MF59 adjuvants, and we optimized immunization strategies including dosage, administration route, and immunization interval in BALB/c mice. Our results demonstrated that a 10 μg dose of inactivated rH5N8/PR8 with MF59 adjuvant, administered intramuscularly twice at 7-day intervals, induced the strongest immune response and effectively protected mice against challenge with wild-type H5N8 AIVs. Since pandemic influenza vaccines typically require tailored vaccination doses and routes specific to their characteristics, this study provides valuable insights for the development of similar vaccine strains with pandemic potential.

## 1. Introduction

In 1996, the H5N1 subtype of highly pathogenic avian influenza virus (HPAIV) was first isolated and identified in Guangdong Province, China. The subsequent identification of the first case of human fatality due to H5N1 infection in Hong Kong in 1997 established it as a virus of significant concern for human health. H5 HPAIVs have undergone rapid genetic diversification and reassortment, resulting in the emergence of multiple clades (clade 0–9) and numerous subclades [1], and have triggered intercontinental transmission events through migratory bird populations. By late 2019, the naming system for H5 subtype was updated, dividing the 2.3.4.4 clade of H5 subtype into eight subclades, 2.3.4.4a through h [2]. Subclade 2.3.4.4b has become the dominant subclade since 2020–2021, primarily comprising the H5N1, H5N6, and H5N8 subtypes. Notably, the H5N8 HPAIV, first detected in poultry and wild birds in the Republic of Korea in 2014, has caused three major epidemic waves (2014–2015, 2017–2018, and 2020–2021) linked to migratory bird activity and global trade. This subtype has spread to all continents, posing a consistent threat to both animal and public health [3,4,5,6,7].

During 2021–2022, clade 2.3.4.4b H5N8 viruses caused an unexpected epidemic peak. In Europe alone, the virus triggered 2467 poultry outbreaks, affecting 477 million birds; there were 187 detections in captive birds and 3573 highly pathogenic avian influenza (HPAI) incidents in wild birds [8]. During this period, the World Health Organization (WHO) also reported the first human infection cases of HPAI H5N8 virus [9]. Concurrently, H5N1 and H5N6 avian influenza viruses (AIVs) were detected in poultry and wild birds, with most belonging to H5 subtype clade 2.3.4.4b along with H5N8 subtype [4,10]. Mutated H5Nx variants triggered broader and more widespread panzootic outbreaks, H5N8 viruses continued evolution and reassortment with other influenza viruses, and the H5N1 subtype exhibited expanded host tropism, including humans [11,12]. A comprehensive analysis of epidemiological, genetic, and bird migration data revealed that a genotypic substitution of the H5N8 viruses (G1 genotype) in 2020 led to the 2021/2022 H5N1 avian influenza outbreaks [11]. Furthermore, H5N8 HPAIVs readily recombine with low pathogenic avian influenza viruses (LPAIVs) to produce different subtypes of H5Nx AIV—including subtypes N1, N2, N5 and N6—that have led to the emergence of new subtypes of avian influenza outbreaks in several countries and regions [7,12,13,14,15,16,17]. The ongoing HPAI outbreaks caused by the H5N8 virus demonstrate that migratory birds are not only natural hosts, but also potential sources of disease outbreaks that generate and spread new variants.

No universal vaccine against the H5 subtype HPAI is currently available globally. China has introduced H5Nx vaccines for avian species [18,19], and clinical trials against the human H5N1 subtype have been conducted in preparation for potential influenza pandemics [2,20,21,22,23]. In addition to the protective effect of the vaccine, the vaccine dose and immunization frequency are also key factors in preventing H5 subtype avian influenza infections. Unlike seasonal influenza virus vaccines, which require only a single dose, studies have reported that due to the low immunogenicity of the hemagglutinin (HA) protein of H5 subtypes, booster immunization is required to increase the protective titers of serum-neutralizing antibodies in vaccinated individuals [24,25,26]. There is antigenic variation within and between H5N1, H5N6, and H5N8 subtypes in subclade 2.3.4.4b, with HA key epitope variants being an important factor. H5N1 virus sera were less reactive with both H5N6 and H5N8 subtypes, and there were antigenic differences between H5N1 and some H5N6 viruses, whereas H5N8 viruses showed fewer differences compared to these two subtypes [27]. This study aimed to evaluate the immunogenicity of a reassortant H5N8 vaccine in a mouse model in terms of antigen dosage, adjuvant, intervals of immunization, and route of administration, as well as to further optimize its immunization regimen. The development of an effective human vaccine platform against potential H5N8 and other H5Nx pandemics has become critical for pandemic preparedness, and this study provides a foundation for building similar vaccine strains with pandemic potential.

## 2. Materials and Methods

### 2.1. Construction of Candidate Vaccine Strain

The HA and Neuraminidase (NA) genes (GISAID accession No. EPI 1846961 and EPI 1846963) were synthesized (Sangon Biotech, Shanghai, China) based on the A/Astrakhan/3212/2020 (H5N8) virus, with the polybasic cleavage site in HA modified from LRERRKRGLF to LRTRGLF (Table S1). Six internal genes (PB1, PB2, PA, NP, M and NS) were derived from the A/Puerto Rico/8/1934 (H1N1) (PR8) virus. All eight genes were cloned into the pHW2000 plasmid vector for rescuing the H5N8 candidate vaccine virus (CVV) by reverse genetics (RG) [28,29], designated as rH5N8/PR8. The reassortant virus was propagated in specific-pathogen-free (SPF) embryonated chicken eggs for 48 h at 37 °C. Allantoic fluid supernatants were harvested and stored at −70 °C until further analysis. Viral infectivity was measured by HA used with 1% equine erythrocytes. The 50% tissue culture infective dose (TCID_50_) and 50% egg infectious dose (EID_50_) were determined in MDCK cells and embryonated eggs, respectively, and calculated using the Reed–Muench method [30]. The rH5N8/PR8 genome was sequenced using next-generation sequencing (NGS) on the Illumina NextSeq 550 Sequencing System platform (Illumina, San Diego, CA, USA) according to the manufacture’s instructions and analyzed using BioEdit (Version 7.1.3.0, Ibis Therapeutics, FL, USA) software [31,32].

### 2.2. Receptor-Binding Preference Assay

The receptor-binding properties of the virus were analyzed using a solid-phase direct binding assay as previously described [33], with two distinct synthetic sialylglycopolymers: α2,3-linked sialic acid (3′-Sialyl-N-acetyllactosamine: Neu5Acα2-3Galβ1-4GlcNAcβ-sp3, 3′SLN) and α2,6-linked sialic acid (6′-Sialyl-N-acetyllactosamine: Neu5Acα2-6Galβ1-4GlcNAcβ-sp3, 6′SLN) (GlycoNZ Corporation, Auckland, New Zealand). Briefly, microtiter plates (Costar 3590) were coated with serial 2-fold dilutions of sialylglycopolymers followed by incubation with 32 HA units of each virus. The plates were subsequently incubated with a mouse anti-H5 monoclonal antibody (mAb) at 37 °C for 1 h and then treated with horseradish peroxidase (HRP)-conjugated goat anti-mouse IgG antibody (Sigma-Aldrich, St. Louis, MO, USA) at 37 °C for 1 h. All samples were analyzed in duplicate, and absorbance was measured at 450 nm using an Epoch Microplate Spectrophotometer (BioTek Instruments, Beijing, China).

### 2.3. Virulence Evaluation of rH5N8/PR8

#### 2.3.1. Intravenous Pathogenicity Test (IVPI)

IVPI was conducted to determine the virulence of rH5N8/PR8 in chickens. Ten 6-week-old SPF chickens were injected intravenously with 100 μL of a 1:10 dilution of rH5N8/PR8 stock solution and monitored for 10 days. During daily observation, chickens were scored according to World Organization for Animal Health (WOAH) Guidelines: 0—if normal, 1—if sick, 2—if severely sick, and 3—if dead. A virus was considered highly pathogenic when the average IVPI value exceeded 1.2 [34].

#### 2.3.2. Virulence of the rH5N8/PR8 in Mice

Female 8-week-old SPF BALB/c mice (18–20 g) were lightly anesthetized with isoflurane and intranasally inoculated with 10^1^–10^7^ TCID_50_ of rH5N8/PR8 in 50 μL phosphate-buffered saline (PBS). Weight changes were monitored daily from 1 to 14 days post-infection and calculated as a percentage of initial weight at day 0 (*n* = 5/group). Mice exhibiting severe sickness with ≥25% weight loss were humanely euthanized. The 50% mouse lethal dose (MLD_50_) was determined using established methods [35]. Clinical signs and body weight changes for each group were recorded daily throughout the 14-day observation period.

### 2.4. Immunization of Mice and Optimization of Vaccination Protocols

To investigate the effects of different adjuvants and optimize the dosage of rH5N8/PR8, 8-week-old female BALB/c mice were randomly assigned to 10 groups (Table S2). These groups included three different doses of inactivated antigen (10 μg, 20 μg, and 40 μg) combined with three adjuvants (ISA51, Alum, and MF59), along with a control group (PBS). All adjuvants were administered according to manufacturers’ instructions (the volume ratio of adjuvant to antigen was 1:1).

The rH5N8/PR8 virus was inactivated with 0.05% β-Propiolactone (β-PPL) for immunization. The suspensions were then purified using a combination of ultra-centrifugation (Beckman Coulter Optima, USA) at 120,000× *g* for 90 min and sucrose density gradient centrifugation (30% and 60% *w*/*v*) to isolate virions from non-viral components. The total protein concentration of the purified inactivated virus was quantified using NanoDrop One^c^ (Thermo Fisher Scientific, Waltham, MA, USA). Inactivated virus solutions containing 10 μg, 20 μg, and 40 μg of protein were prepared in 50 μL of PBS for subsequent experiments.

To evaluate the appropriate dosage, each mouse received an intramuscular injection of 100 μL candidate vaccine rH5N8/PR8 containing either 10 μg, 20 μg, or 40 μg of inactivated antigen, followed by a booster immunization. Serum samples were collected via retro-orbital venous plexus on days 0, 7, 14, and 28 after primary immunization to measure hemagglutination inhibition (HI) antibody titers.

We further investigated the impact of immunization routes on immune responses (Table S3). BALB/c mice were immunized via intramuscular (i.m.) or subcutaneous (s.c.) routes with 10 μg of inactivated rH5N8/PR8 antigen formulated with MF59 adjuvant, followed by a booster dose. Serum samples were collected via retro-orbital venous plexus on days 0, 7, 14, and 28 post-primary immunization to assess HI antibody titers.

The effect of immunization intervals was also analyzed (Table S4). Mice received 10 μg of inactivated antigen combined with MF59 adjuvant via intramuscular injection, with booster immunizations administered at different intervals (7, 14, or 21 days). Serum was collected on days 0, 7, 14, and 28 after the initial immunization for HI antibody titer determination.

Finally, long-term immunological observations were conducted over 24 weeks (Table S5). Mice received 10 μg of inactivated antigen combined with different adjuvants (ISA51, Alum, MF59) via intramuscular injection at 7-day intervals. Serum samples were collected via retro-orbital venous plexus at 7 d, 14 d, 28 d, 6 w, 8 w, 12 w, 18 w, and 24 w after primary immunization to evaluate HI antibody titers.

### 2.5. Testing of Immunoglobulin and Cytokines in Sera

Immunoglobulin antibody titers were quantified using a standardized enzyme-linked immunosorbent assay (ELISA) according to established protocols [35]. The antibody-positive threshold was defined as the mean + 2 × SD of values derived from control group measurements. For multiplex cytokine profiling, serum samples were analyzed using the Luminex xMAP^®^-based Multi-Cytokine Detection Kit (EPX140-15815-901; Leitz Biotechnology Co., Ltd., Beijing, China) on a Luminex^®^ 200™ system (Luminex Corporation, Austin, TX, USA) [36]. Experimental procedures strictly followed the manufacturer’s technical manual, including standard curve generation through five-parameter logistic (5-PL) nonlinear regression modeling. All analyte concentrations were interpolated from triplicate measurements with inter- and intra-assay coefficients of variation <15%.

### 2.6. Protective Efficacy Evaluation of H5N8 Vaccine in BALB/c Mice

**Figure 1:**
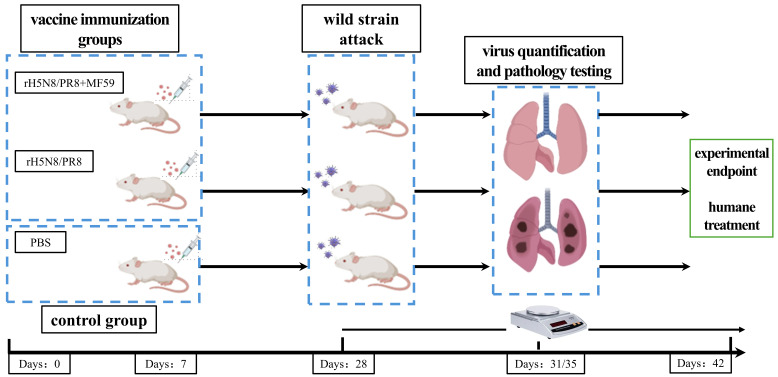
Protocol design for evaluating the protective efficacy of rH5N8/PR8 in a mouse model. BALB/c mice were randomly allocated into two vaccine immunization groups and a control group. The vaccination groups received two intramuscular injections of 10 μg inactivated rH5N8/PR8 (with or without MF59 adjuvant) at 7-day intervals, while the control group was injected with PBS. On day 28 post-primary immunization, mice were challenged intranasally with wild-type WT13404 (H5N8). Lung and tracheal tissues were collected on days 3 and 5 post-challenge for pathogenicity analysis.

Female BALB/c mice (8 weeks old) were randomly allocated into three experimental groups (*n* = 15/group) (Table S6). Two vaccination groups received two doses of intramuscular injections of rH5N8/PR8 candidate vaccine at a 7-day interval, with doses containing either 10 μg inactivated viral antigen in 50 μL PBS with MF59 adjuvant (50 μL) or the equivalent antigen without adjuvant. The control group received 100 μL sterile PBS per mouse (Figure 1).

On day 28 post-primary immunization, mice were anesthetized via isoflurane inhalation and intranasally challenged with the wild-type A/Swan/Inner Mongolia/13404/2020 (H5N8) (abbreviated as WT13404) virus at 10 MLD_50_ (corresponding to 10^2.5^ TCID_50_/50 μL). Clinical monitoring included daily recording of body weight changes and survival rates for 14 days, with ≥25% weight loss defined as the humane endpoint.

For pathogenesis analysis, five mice in each group were sacrificed by cervical dislocation at days 3 and 5 post-challenge. Complete necropsy was performed to collect lung and tracheal tissues, which were fixed in 4% paraformaldehyde for histopathological evaluation. Hematoxylin & eosin (H&E) staining and immunohistochemistry (IHC) were conducted using monoclonal anti-influenza A nucleoprotein antibody (Abcam Cat# ab20841, Waltham, MA, USA, 1:500 dilution) with HRP-conjugated secondary antibody (Dako EnVision+System).

### 2.7. Biosafety and Animal Ethics

Female BALB/c mice (8 weeks old) used in this study were obtained from Beijing Vital River Laboratory Animal Technology Co., Ltd. (Beijing, China) and maintained under SPF conditions with sterile feed and water. All experimental procedures were conducted in either biosafety level 2 (BSL-2) or BSL-3 animal laboratories. The animal experiments were approved by the Animal Experimental Ethical Inspection Committee of the National Institute for Viral Control and Prevention, China CDC (Approval Nos.: 20220315037, and 20220727086) and were performed in accordance with strict approved protocols.

## 3. Results

### 3.1. Construction of H5N8 CVV

Full genome sequencing confirmed that the HA and NA genes of rH5N8/PR8 were identical to A/Astrakhan/3212/2020 (H5N8), except for the deletion of polybasic cleavage sites associated with high pathogenicity in HA (Table S1). The six internal segments (PB1, PB2, PA, NP, M, NS) were derived from A/Puerto Rico/8/1934 (H1N1) (PR8). Hemagglutination testing with 1% equine erythrocyte suspensions demonstrated an HA titer of 2^8^ (256 HAU/50 μL). Genetic stability was assessed through 10 serial passages (designated E1–E10) in SPF-embryonated chicken eggs, with whole-genome deep sequencing revealing consistent sequence identity (100% nucleotide similarity, with 90% cutoffs for consensus sequences) between ten passages. We have submitted all the sequences to GASID, and the accession numbers are from EPI_ISL_19805753 to EPI_ISL_19805762. The EID_50_ value was 10^−9^/mL, and the TCID_50_ titer was 10^−6^/50 μL.

### 3.2. Receptor-Binding Preference Analysis

**Figure 2:**
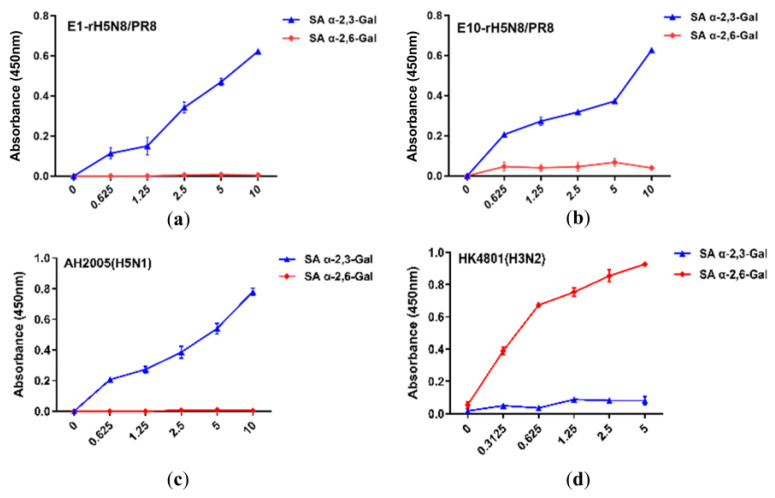
Characterization of the receptor-binding properties of rH5N8/PR8. Binding affinity to two different biotinylated glycans (3′SLN, 6′SLN) was tested. SA α-2,3-Gal and SA α-2,6-Gal were used to represent 3′SLN and 6′SLN, respectively. Both E1 (**a**) and E10 (**b**) passages of rH5N8/PR8 demonstrated binding to 3′SLN at concentrations of 0.625, 1.25, 2.5, 5, and 10 μg/mL. The data presented are means from two separate assays performed in duplicate, with absorbance measured at 450 nm. A/Anhui/2005(H5N1) virus (which preferentially binds to SA α-2,3-Gal) and seasonal influenza virus A/Hong Kong/4801/2014(H3N2) (which preferentially binds to SA α-2,6-Gal) were used as controls (**c**,**d**). AH2005 represents A/Anhui/2005, HK4801 represents A/Hong Kong/4801/2014.

Receptor-binding properties analysis revealed that the rH5N8/PR8 vaccine candidate maintained specific binding affinity to avian-type receptors (SA α2,3-Gal) while showing no detectable binding to human-type receptors (SA α2,6-Gal). Importantly, serial passages in embryonated chicken eggs through 10 generations (E1–E10) demonstrated stable preservation of the avian virus-like receptor tropism, with both early (E1) and late (E10) passage viruses retaining identical binding profiles to the parental strain (Figure 2). Based on rH5N8/PR8′s specific binding affinity to SA α2,3-Gal, intramuscular and subcutaneous administration routes were selected instead of respiratory immunization.

### 3.3. Pathogenicity Assessment

The IVPI evaluation was conducted on ten 6-week-old SPF chickens. Throughout the 10-day monitoring period, no clinical symptoms were observed in any of the chickens. The calculated IVPI score was 0, demonstrating that rH5N8/PR8 is a low-pathogenicity strain.

**Figure 3:**
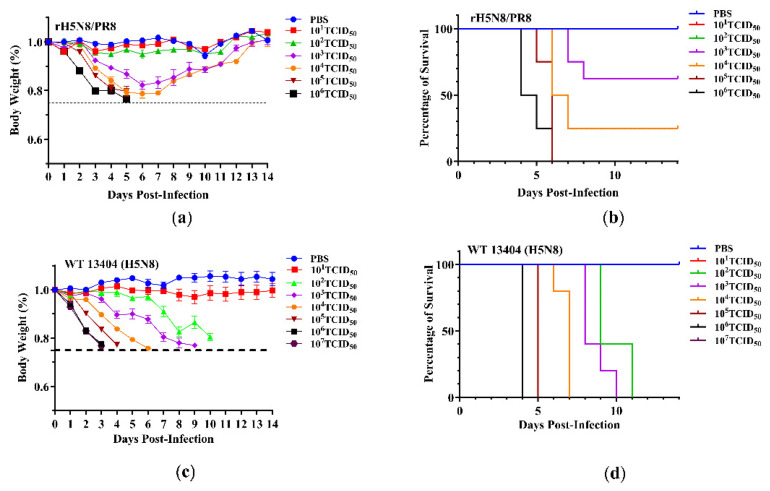
Pathogenicity analysis of rH5N8/PR8. BALB/c mice lightly anesthetized by isoflurane were intranasally inoculated 10^1^–10^6^ TCID_50_ of rH5N8/PR8 or 10^1^–10^7^ TCID_50_ wild-type H5N8. Weight change (**a**) and survival curve (**b**) of mice infected with rH5N8/PR8; weight change (**c**) and survival curve (**d**) of mice infected with WT13404 (H5N8).

Virulence was further evaluated by determining the MLD_50_ in a mouse model. Groups of BALB/c mice (*n* = 5 per group) were intranasally administered 10-fold serial dilutions of either rH5N8/PR8 or the wild-type WT13404 (H5N8). Body weight and survival rates were monitored daily for 14 days post-inoculation (Figure 3). rH5N8/PR8 exhibited significantly attenuated virulence, with an MLD_50_ of 10^3.39^ TCID_50_ compared to 10^1.5^ TCID_50_ for WT13404 (H5N8). Although this 100-fold difference in MLD_50_ indicates substantial attenuation of rH5N8/PR8 in mice through intranasal administration, an inactivated vaccine was deemed more suitable for safety considerations and was therefore used for the subsequent immunogenicity and protective efficacy studies.

### 3.4. Immunogenicity and Optimization of Immunization Protocols for rH5N8/PR8 Candidate Vaccine

#### 3.4.1. Immunogenicity Effect of Different Adjuvants and Dosage

**Figure 4:**
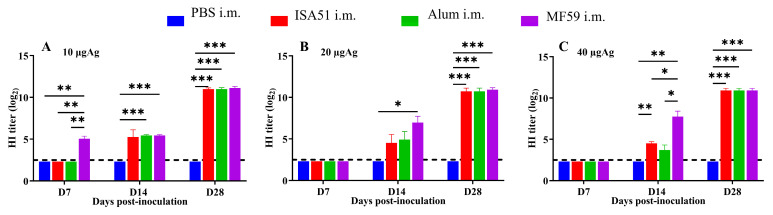
Immunogenicity effects of different adjuvants and dosage. HI antibody induced by adjuvanted vaccines via intramuscular injection at 7-day intervals. (**A**) 10 μg inactivated rH5N8/PR8 vaccine with different adjuvants (ISA51, Alum, and MF59); (**B**) 20 μg inactivated rH5N8/PR8 vaccine with different adjuvants; (**C**) 40 μg inactivated rH5N8/PR8 vaccine with different adjuvants. *: *p* < 0.05, **: *p* < 0.01, ***: *p* < 0.001.

**Figure 5:**
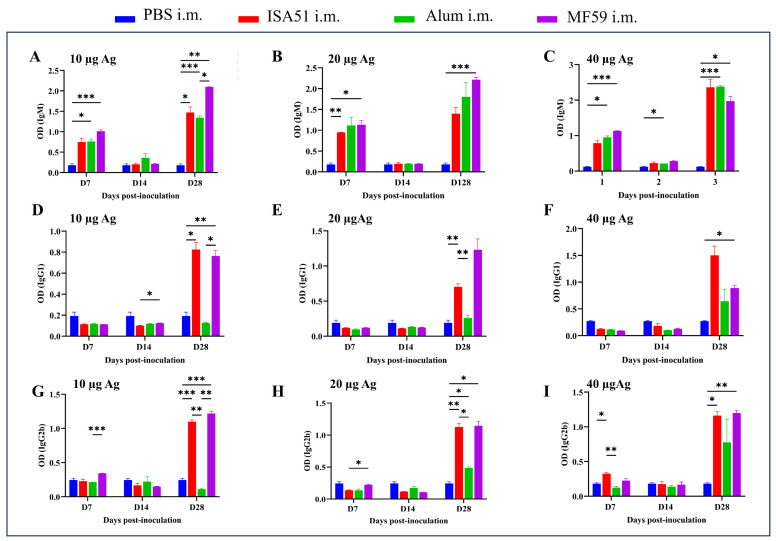
Immunoglobulin (Ig) induced by rH5N8/PR8 in sera of mice. Ig antibody titers were induced by inactivated rH5N8/PR8 vaccine with different doses (10 μg, 20 μg, or 40 μg) and adjuvants (Alum, MF59, or ISA51) via intramuscular injection at 7-day intervals, and quantified using a standardized ELISA: IgM (**A**–**C**), IgG1 (**D**–**F**), and IgG2b (**G**–**I**). *: *p* < 0.05, **: *p* < 0.01, ***: *p* < 0.001.

**Figure 6:**
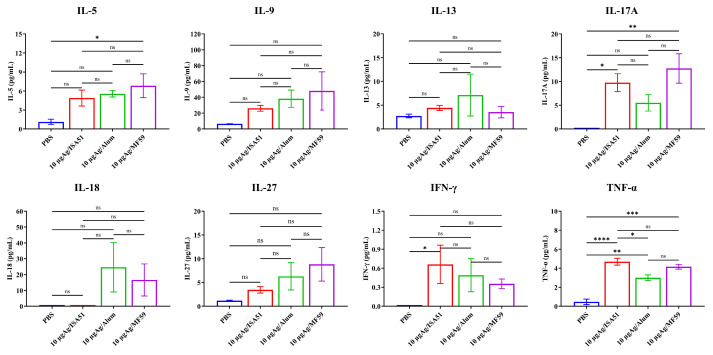
Testing of cytokines induced by rH5N8/PR8 in sera of mice. Cytokines were induced by 10 μg inactivated rH5N8/PR8 vaccine with MF59 adjuvant via intramuscular injection at 7-day intervals. Serum samples were analyzed using standard curve generation through five-parameter logistic nonlinear regression modeling. All analyte concentrations were interpolated from triplicate measurements with inter- and intra-assay coefficients of variation <15%. The concentrations of all samples were calculated as the mean based on three repeated measurements. ns: not significant, *: *p* < 0.05, **: *p* < 0.01, ***: *p* < 0.001, **** *p* < 0.0001.

To determine the optimal immunization parameters, SPF female BALB/c mice (8 weeks old, 18–20 g) were immunized intramuscularly with inactivated rH5N8/PR8 formulated with different adjuvants. The adjuvanted vaccine groups (Alum, MF59, and ISA51) elicited significantly higher antibody responses compared to non-adjuvanted controls, with HI antibody titers first detected on day 7 post-primary immunization. Quantitative analysis revealed that the MF59-adjuvanted formulation induced superior HI antibody titers at all time points (days 7, 14, and 28 post-immunization), outperforming all other adjuvant groups (Figure 4A–C). This formulation also elicited significantly elevated IgM levels on days 14 and 28 after boost immunization (*p* < 0.001 vs. other groups; Figure 5A–I). Cytokines IL-5, IL-17A, and TNF-α were detected at higher levels in the 10 μg MF59-adjuvanted formulation group compared to PBS groups (*p* < 0.05, *p* < 0.01, *p* < 0.001, respectively). Cytokines, including IFN-γ, IL-9, IL-13, IL-18, and IL-27, were not detected significantly differently in different groups (Figure 6).

#### 3.4.2. Immunogenicity Difference Between Intramuscular and Subcutaneous Administration

**Figure 7:**
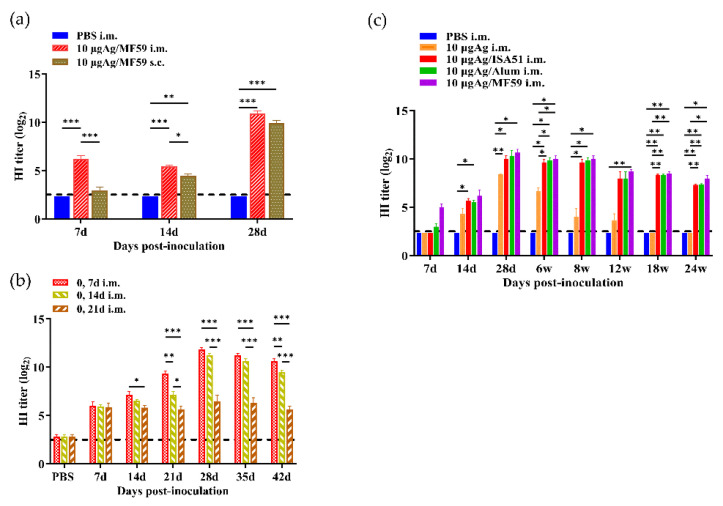
Comparison of different immunization strategies of rH5N8/PR8. (**a**) HI antibody induced by 10 μg inactivated rH5N8/PR8 vaccine with MF59 adjuvant via different immune routes (intramuscular injection, subcutaneous injection) at 7-day intervals; (**b**) HI antibody induced by 10 μg inactivated rH5N8/PR8 vaccine with MF59 adjuvant via intramuscular injection at 7-day, 14-day and 21-day intervals; (**c**) Results of long-term immune response of inactivated rH5N8/PR8 vaccine with different adjuvants. *: *p* < 0.05, **: *p* < 0.01, ***: *p* < 0.001.

Twenty-four female SPF BALB/c mice (8 weeks old) were randomly divided into three groups with eight animals per group (Table S3). The experimental groups included one intramuscular injection group and one subcutaneous injection group, both receiving a mixture containing 10 μg of rH5N8/PR8 vaccine strain and 50 μL of MF59 adjuvant. The control group received 100 μL of PBS via intramuscular injection as a negative control. All experimental groups received a booster immunization on day 7 after the initial vaccination, maintaining identical vaccine dosage and administration route. Blood samples were collected via orbital venous plexus puncture on days 7, 14, and 28 post-initial immunization, and serum was isolated by centrifugation to test specific HI antibody titers using the HI assay. A comparative evaluation of administration routes demonstrated that intramuscular delivery of the 10 μg MF59-adjuvanted vaccine generated significantly higher HI titers than subcutaneous administration throughout the 28-day observation period (*p* < 0.01) (Figure 7a). Based on these findings, intramuscular delivery was selected for subsequent challenge experiments.

#### 3.4.3. Comparison of Immunogenicity at Different Booster Intervals

This study further explored the influence of different intervals between priming and boosting on the immune response. Thirty-two female SPF BALB/c mice (8 weeks old) were randomly divided into four groups (*n* = 8 per group) (Table S4). Three prime-boost interval schedules were implemented: 7-day, 14-day, and 21-day intervals. All groups received two intramuscular vaccinations with identical antigen doses (10 μg rH5N8/PR8 + 50 μL MF59 adjuvant). Blood samples were collected via orbital venous plexus puncture on days 7, 14, and 28 post-prime immunization. Serum was analyzed for HI antibody titers. Comparative evaluation of different immunization intervals demonstrated that the 7-day intervals generated significantly higher HI titers than the 14-day and 21-day intervals throughout the 42-day observation period (Figure 7b). Based on these findings, the optimized formulation with a 7-day booster interval was selected for subsequent challenge experiments.

#### 3.4.4. Long-Term Immune Response Monitoring

A 24-week longitudinal immunological study was established to evaluate antibody persistence (Table S5). Forty female SPF BALB/c mice (8 weeks old, 18–20 g) were randomly divided into five groups (*n* = 8/group) and immunized intramuscularly. All groups received prime and booster vaccinations (7-day interval between doses) with 10 μg inactivated antigen, formulated either alone or in combination with ISA51, Alum, or MF59 adjuvants. Serum samples were collected via retro-orbital venous plexus at specified time points: 7 d, 14 d, 28 d, 6 w, 8 w, 12 w, 18 w, and 24 w post-prime immunization. Specific HI antibody titers were quantitatively determined using standardized HI assays. Long-term immune monitoring demonstrated sustained HI antibody responses for at least 24 weeks post-primary immunization across all adjuvant groups with one booster immunization at day 7, with the MF59-adjuvanted formulation maintaining the highest HI antibody titers at all points in time (6, 8, 12, 18 and 24 weeks) (Figure 7c).

A 24-week longitudinal immunological study was established to evaluate antibody persistence (Table S5). Forty female SPF BALB/c mice (8 weeks old, 18–20 g) were randomly divided into five groups (*n* = 8/group) and immunized intramuscularly. All groups received prime and booster vaccinations (7-day interval between doses) with 10 μg inactivated antigen, formulated either alone or in combination with ISA51, Alum, or MF59 adjuvants. Serum samples were collected via retro-orbital venous plexus at specified time points: 7 d, 14 d, 28 d, 6 w, 8 w, 12 w, 18 w, and 24 w post-prime immunization. Specific HI antibody titers were quantitatively determined using standardized HI assays. Long-term immune monitoring demonstrated sustained HI antibody responses for at least 24 weeks post-primary immunization across all adjuvant groups with one booster immunization at day 7, with the MF59-adjuvanted formulation maintaining the highest HI antibody titers at all points in time (6, 8, 12, 18 and 24 weeks) (Figure 7c).

### 3.5. Evaluation of Protective Efficacy for MF59-Adjuvanted Inactivated rH5N8/PR8

**Figure 8:**
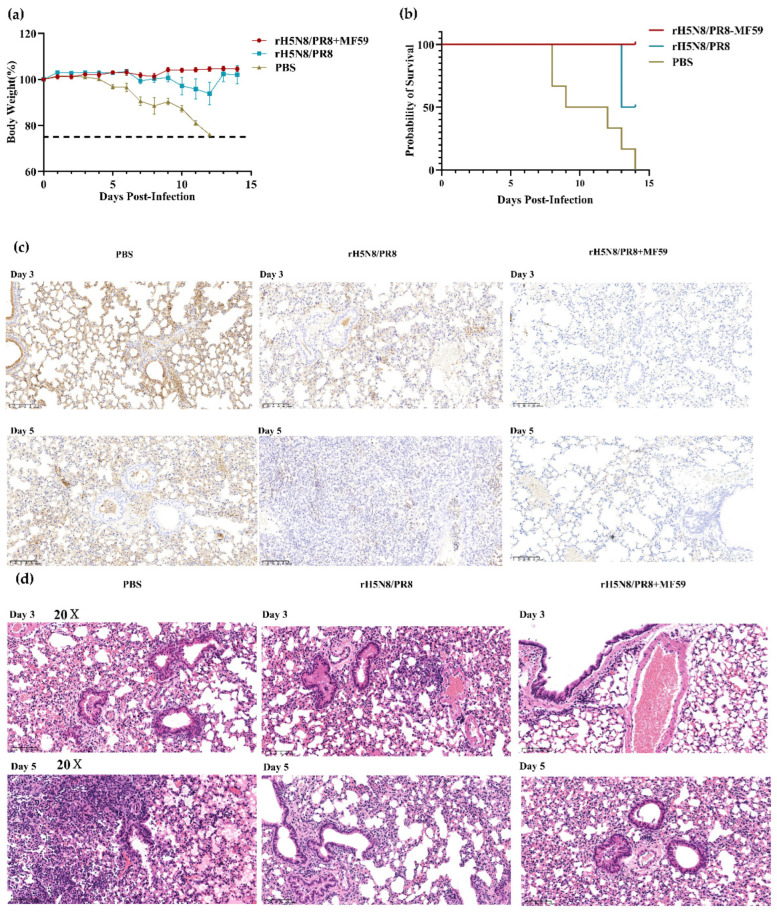
Evaluation of protective efficacy for MF59-adjuvanted inactivated rH5N8/PR8. Weight changes (**a**) and survival curve (**b**) of mice immunized after challenge. (**c**) IHC detection of viral nucleoprotein at days 3 and 5 post-challenge (200×). Diffuse positive staining observed in control bronchioles versus focal positivity in vaccinated lungs; (**d**) H&E-stained lung sections at days 3 and 5 post-challenge (200×). Control group exhibits marked alveolar consolidation and peribranchial lymphocytic infiltration. Vaccinated group maintains normal alveolar architecture.

The mice were challenged intranasally with 10 × MLD_50_ of WT13404 (H5N8) (50 μL/mouse) 28 days after boost immunization. The immunization protocol consisted of two intramuscular administrations (7-day interval) of 10 μg inactivated rH5N8/PR8 antigen formulated with 50 μL MF59 adjuvant. In the control group, progressive weight loss began on day 5 post-challenge, resulting in 100% mortality between days 8–13. Notably, the MF59-adjuvanted vaccine group conferred complete protection against lethal challenge (0% mortality), whereas the non-adjuvanted vaccine group provided only partial protection (60% survival rate) (Figure 8a,b).

IHC results revealed numerous positive cells in the bronchial epithelium, alveolar epithelial cells, and alveolar cavities of the control PBS group. In contrast, the adjuvant immunization group showed fewer positive cells that were localized to specific areas (Figure 8c). H&E staining demonstrated extensive inflammatory cell infiltration in the alveolar walls of control animals. This widespread consolidation of alveolar walls led to collapse of the alveolar structure. The alveolar cavities contained numerous inflammatory cells, along with small amounts of necrotic cell debris and red blood cells. These pathological changes indicate that, compared to the immunized group, the PBS control group exhibited significantly more severe inflammatory responses and tissue damage (Figure 8d).

## 4. Discussion

AIVs pose a persistent threat to wild birds, poultry, and mammals, including humans. Following the 2014 outbreak of HPAIV H5N8 in South Korea, H5N8 and H5N1 subtypes of clade 2.3.4.4b have triggered a global epidemic, resulting in substantial economic losses, ecological disruption, and significant public health challenges. Notably, seven human cases of H5N8 influenza virus infection were reported in the Russian Federation in 2020. Previous studies suggest that H5N8 viruses not only contribute internal gene segments to other H5Nx subtypes, but also readily undergo reassortment with LPAIVs [5,6,13]. Such genetic exchanges may generate novel recombinant H5Nx variants with zoonotic potential. Hill et al. demonstrated that poultry populations facilitate the slow but sustained spread of H5 subtype HPAIV outbreaks, whereas wild birds (e.g., swans and gulls) mediate rapid, intermittent transmission through distinct migratory routes [37]. The intercontinental dissemination of H5 subtype AIVs is strongly associated with the movement patterns of migratory birds [13]. It is particularly important to monitor the evolution of H5N8 subtype clade 2.3.4.4b viruses. Although the H5N8 subtype has not yet demonstrated a capacity for human-to-human transmission, its continuous global circulation, the long-distance migration of wild birds, and the high frequency of antigenic variation and recombination with other LPAIVs present dangers that cannot be ignored. Vaccination remains one of the most effective preventive measures against influenza viruses. However, influenza viruses are susceptible to genetic mutation and recombination between different subtypes under natural evolutionary and selective pressures, often resulting in mismatches between seasonal influenza vaccines and circulating strains [38,39]. This reduces vaccine effectiveness, particularly in vulnerable populations, such as the elderly, patients with serious diseases, and young children [40]. Currently, no universal vaccine exists for H5 subtype HPAIV globally, although phase I clinical trials for human H5N1 vaccines have been conducted in several countries in preparation for potential influenza pandemics.

While annual single-dose vaccination is sufficient for seasonal influenza, pandemic influenza vaccine development faces challenges in determining optimal administration routes, dosages, and immunization schedules. Immunogenicity and adjuvant selection are key factors in preventing H5 subtype influenza virus infections. Unlike seasonal influenza vaccines that require only one dose, enhanced immunization protocols are needed to improve protective antibody titers due to the low immunogenicity of H5 subtype HA. Numerous studies have demonstrated that H5N1-inactivated vaccines often require adjuvants to reduce antigen quantities and enhance protective immunity. In this study, we first used reverse genetics technology to construct a human pandemic vaccine candidate virus from the clade 2.3.4.4b H5N8 subtype. Through in vivo animal models, we systematically evaluated the proliferation characteristics, virulence level, and immunological safety of the rH5N8/PR8 vaccine strain to provide a theoretical basis for its development as a candidate vaccine. The HA gene of H5N8 virus was modified to reduce pathogenicity. The IVPI experiment confirmed that the rH5N8/PR8 candidate virus was a low pathogenic strain. Growth curve measurements showed that rH5N8/PR8 could replicate effectively in mammalian cells, with slightly higher replication in MDCK cells compared to the PR8 strain. The vaccine strain rH5N8/PR8 constructed in this study effectively protected mice against challenge with wild-type H5N8 virus. Our study demonstrated that the optimal immunization strategy for the rH5N8/PR8 vaccine is a 10 μg dose of inactivated rH5N8/PR8 combined with 50 μL ofMF59 adjuvant administered intramuscularly with a booster immunization at 7-day intervals. This regimen induced the strongest immune response and effectively protected BALB/c mice against challenge with wild-type H5N8 AIV.

Most clinical studies consistently demonstrate that avian influenza vaccines exhibit lower immunogenicity and weaker immune memory in humans compared to seasonal influenza vaccines, often necessitating adjuvanted formulations and multi-dose regimens to enhance efficacy. Adjuvant-mediated immune enhancement is well-documented, with studies confirming elevated HI antibody titers post-vaccination [41,42,43]. Among clinically validated adjuvants, MF59 and Alum have been extensively characterized for safety and efficacy in human trials, while ISA51 remains under investigation for influenza applications. Gao et al. compared the immune effects of different doses and different adjuvants (AddaVax and Al(OH)3) on the H5N8-inactivated vaccine [44]. Parallel findings by Winokur et al. in a randomized blinded trial revealed that two doses of AS03- or MF59-adjuvanted H5N8 vaccine (7.5 or 15 μg HA) induced strong homologous immunogenicity, but demonstrated limited cross-reactivity against antigenically drifted H5N1 strains [45]. The vaccine constructed in this study is not a broad-spectrum vaccine; it provides protection against viruses with similar antigenicity in the same clade, but it cannot provide effective protection against viruses in other clades, such as clade 2.3.2.1. Such vaccines have better specificity compared to broad-spectrum vaccines. Therefore, we did not conduct cross-protective experiments on other clades of H5 virus. The vaccine strain in this study was based on the only human isolates of H5N8 subtype virus and, although 5 years have passed, H5 viruses with these genetic and antigenic characteristics still circulate in birds [46]. This study has determined the optimal adjuvant, immunization route, dose, and interval for the H5N8 subtype vaccine strain, providing a scientific basis for the construction of similar vaccine strains. The H5N8 subtype vaccine strain constructed in this study can provide technical and platform support for the development of pandemic vaccines for human use.

## Data Availability

Data supporting the findings of this study are included within the article.

## Supplementary Materials

The following supporting information can be downloaded at: https://www.mdpi.com/article/10.3390/v17040544/s1, Table S1: Modification of Hemagglutinin Cleavage Site in A/Astrakhan/3212/2020 (H5N8); Table S2: Grouping of the effects of different adjuvants and antigen doses on the immune response in a mouse model; Table S3: Grouping of the effects of different immunization routes on the immune response in a mouse model; Table S4: Grouping of the effects of different immunization intervals on the immune response in a mouse model; Table S5: Grouping for long-term monitoring of different adjuvants (ISA51, Alum, and MF59) on the immune response in a mouse model; Table S6: Grouping for protective efficacy evaluation of rH5N8/PR8 candidate vaccine in a mouse model.
