# Supplementary material for: Construction and Immune Strategy Optimization of a Vaccine Strain for Influenza A (H5N8) Subtype
Source: Viruses. 2025 Apr 8;17(4):544. doi: 10.3390/v17040544 (PMC12031352; doi:10.3390/v17040544)
Supplement: Supplementary file 1 [file viruses-17-00544-s001.zip › viruses-3521840-supplementary.pdf]

Supplementary Table S1. Modification of Hemagglutinin Cleavage Site in A/Astrakhan/3212/2020 (H5N8).

A/Astra-khan/3212/2020 (H5N8)	Modification of Hemagglutinin Cleavage Site									
Wild virus HA	L<br>AGA	R<br>GAA	E<br>AAG	R<br>AGA	R<br>AGA	K<br>AAA	R<br>AGA	↓G<br>GGC	L<br>CTG	F<br>TTT
Modified HA	AGA<br>L	GAA<br>R	AAG<br>T	AGA<br>R	–	–	–	GGC<br>G	CTG<br>L	TTT<br>F

↓.Proteolytic cleavage site.

Supplementary Table S2. Grouping of the effects of different adjuvants and antigen doses on the immune response in a mouse model.

Group	rH5N8/PR8 (per mice)	Ajuvant (per mice)	BALB/c (N)	Immunization Route	Immunization Interval
group 1	10 μg	50 μL/ISA51	8	i.m.	Day 0, 7
	10 μg	50 μL/Alum	8	i.m.	Day 0, 7
	10 μg	50 μL/MF59	8	i.m.	Day 0, 7
group 2	20 μg	50 μL/ISA51	8	i.m.	Day 0, 7
	20 μg	50 μL/Alum	8	i.m.	Day 0, 7
	20 μg	50 μL/MF59	8	i.m.	Day 0, 7
	40 μg	50 μL/ISA51	8	i.m.	Day 0, 7
group 3	40 μg	50 μL/Alum	8	i.m.	Day 0, 7
	40 μg	50 μL/MF59	8	i.m.	Day 0, 7
group 4 (control)	PBS (100 μL)	–	8	i.m.	Day 0, 7

Supplementary Table S3. Grouping of the effects of different immunization routes on the immune response in a mouse model.

Group	rH5N8/PR8 (per mice)	Adjuvant (per mice)	BALB/c (N)	Immunization Route	Immunization Interval
group 5	10 μg	50 μL/MF59	8	i.m.	Day 0, 7
group 6	10 μg	50 μL/MF59	8	s.c..	Day 0, 7
group 7 (control)	PBS (100 μL)	–	8	i.m.	Day 0, 7

Supplementary Table S4. Grouping of the effects of different immunization intervals on the immune response in a mouse model.

Group	rH5N8/PR8 (per mice)	Adjuvant (per mice)	BALB/c (N)	Immunization Route	Immunization Interval
group 8	10 μg	50 μL/MF59	8	i.m.	Day 0, 7
group 9	10 μg	50 μL/MF59	8	i.m.	Day 0, 14
group 10	10 μg	50 μL/MF59	8	i.m.	Day 0, 21
group 11 (control)	PBS (100 μL)	–	8	i.m.	Day 0, 7

Supplementary Table S5. Grouping for long-term monitoring of different adjuvants (ISA51, Alum and MF59) on the immune response in a mouse model.

Group	rH5N8/PR8 (per mice)	Adjuvant (per mice)	BALB/c (N)	Immunization Route	Immunization Interval
group 12	10 μg	–	8	i.m.	Day 0, 7
group 13	10 μg	50 μL/ISA51	8	i.m.	Day 0, 7
group 14	10 μg	50 μL/Alum	8	i.m.	Day 0, 7
group 15	10 μg	50 μL/MF59	8	i.m.	Day 0, 7
group 16 (control)	PBS (100 μL)	–	8	i.m.	Day 0, 7

Supplementary Table S6. Grouping for protective efficacy evaluation of rH5N8/PR8 candidate vaccine in a mouse model.

Group	rH5N8/PR8 (per mice)	Adjuvant (per mice)	BALB/c (N)	Immunization Route	Immunization Interval
group 17	10 µg	50 µL/MF59	8	i.m.	Day 0, 7
group 18	10 µg	50 µL/MF59	8	i.m.	Day 0, 7
group 19	10 µg	50 µL/MF59	8	i.m.	Day 0, 7
group 20 (control)	PBS (100 µL)	–	8	i.m.	Day 0, 7
